# Age structure is critical to the population dynamics and survival of honeybee colonies

**DOI:** 10.1098/rsos.160444

**Published:** 2016-11-16

**Authors:** M. I. Betti, L. M. Wahl, M. Zamir

**Affiliations:** 1 Department of Applied Mathematics, Western University, London, Ontario, Canada N6A 5B7; 2 Department of Medical Biophysics, Western University, London, Ontario, Canada N6A 5B7

**Keywords:** honeybee colony dynamics, honeybee colony age structure, reproduction number, spring dwindle, honeybee survival

## Abstract

Age structure is an important feature of the division of labour within honeybee colonies, but its effects on colony dynamics have rarely been explored. We present a model of a honeybee colony that incorporates this key feature, and use this model to explore the effects of both winter and disease on the fate of the colony. The model offers a novel explanation for the frequently observed phenomenon of ‘spring dwindle’, which emerges as a natural consequence of the age-structured dynamics. Furthermore, the results indicate that a model taking age structure into account markedly affects the predicted timing and severity of disease within a bee colony. The timing of the onset of disease with respect to the changing seasons may also have a substantial impact on the fate of a honeybee colony. Finally, simulations predict that an infection may persist in a honeybee colony over several years, with effects that compound over time. Thus, the ultimate collapse of the colony may be the result of events several years past.

## 1. Introduction

As honeybee populations continue to decline on a global scale [1], research efforts have been directed at identifying the underlying causes [2–5]. These efforts are necessitated by the ecological [6] and economical importance of honeybees worldwide [7–9]. To date, much of this research has been focused on the effects of pesticide and insecticide exposure on honeybee health and ultimately colony fitness [4,10]. Such hazards may cause injury or death to foraging bees, forcing surviving bees to begin foraging prematurely which will then disrupt the dynamics of the colony, ultimately leading to colony collapse [10–12]. Further research has focused on the effects of parasitism and disease [13–18], for example, the effects of *Varroa destructor* on honeybee colony dynamics [15–18], the effects of the microsporidian parasite *Nosema ceranae* [19] and the effects of communicable infections more generally [13].

Recent work has also explored the combined effects of two stressors such as disease, limited biodiversity and/or exposure to pesticides, which may create conditions detrimental to honeybee colony survival [20–25]. Simulation packages have been developed to model these effects using realistic parameter values [26,27]. For example, Pettis *et al*. [28] explored the interplay between environmental hazards and the subsequent susceptibility to *Nosema ceranae*. In previous work [13], we showed that the fate of a honeybee colony in the presence of disease is dependent on seasonality, particularly the onset of winter.

Previous mathematical models of honeybee dynamics have not considered the effects of age structure within the colony, yet the duties of female bees, which constitute the main work force of the hive, are determined primarily by their age [29]. Specifically, the structure of a colony hinges on both bee morphology and age [30–32]: caste polyethism differentiates a queen bee from a female worker bee [33], while age polyethism determines the functions of the worker bees [29].

Thus, the age distribution within a honeybee colony is an intrinsic property and this property is therefore important to colony survival, because it affects two key components in the dynamics of the colony, namely ongoing recruitment and death [29]. In this paper, we examine how the disease-free age distribution within a colony is altered in the face of a hazard, and how these changes affect previous predictions of colony survival.

We identify periods of increased vulnerability of the colony to the effects of winter, disease or a combination of the two, during which the colony would benefit from remedial actions (e.g. higher anti-microbial treatments, increased observation and management, etc.). In addition, we use the added dimension of age structure to explore the long-standing phenomenon of ‘spring dwindle’ whereby a drop in the number of bees within a colony occurs immediately after the end of winter [34]. While the phenomenon is generally suspected to be due to various stressors [35–37], the specific colony dynamics that lead to this phenomenon have not been established. The question of why the dwindle occurs after rather than during winter remains unanswered. The resolution of this puzzle is of particular value in the ongoing research efforts to understand and ultimately avert honeybee colony collapse, since spring dwindle leaves the colony in a particularly vulnerable state. Finally, we simulate colony dynamics in the presence of disease for multiple years to demonstrate the compounding effects of infection.

## 2. Model

We present a mathematical model that combines the disease-free demographics of a honeybee colony with the effects of seasonal changes and a disease that at first infects foragers, and then spreads to the rest of the colony. Earlier versions of this model were introduced by Khoury *et al.* in 2011 and 2013 [11,12] and were developed further in 2014 [13]. Analytical details of the model, including local and global stability of the disease-free equilibrium as well as a derivation of the basic reproduction number, *R*_0_, are presented in [14].

Briefly, the effects of the brood, guarding bees, as well as bees that work to repair the hive are neglected. The focus is solely on the remaining adult hive bees, *H*, which are responsible for ensuring the survival of the brood, and the foragers, *F*, which are responsible for bringing food, *f*, into the hive. The male honeybees, known as drones, are also neglected since they contribute only to reproduction [33]. In the presence of disease, the two classes of bees are further divided into the susceptible (disease-free) populations, *H*_S_ and *F*_S_, and the infected populations, *H*_I_ and *F*_I_.

### 2.1. Age structure

We incorporate an age structure into the age-independent equations [13], using the standard approach of McKendrick [38] by writing

2.1 $$\frac{\partial H_{S}}{\partial t}+\frac{\partial H_{S}}{\partial a}=-u(a)H_{S}-\beta NH_{S},$$

where *H*_S_(*a*,*t*) is the number of susceptible hive bees of age *a* at time *t* and *u*(*a*) is the age-dependent rate of recruitment to foraging. Juvenile hormone III regulates the age at which honeybees begin foraging [29], making older bees more likely to be recruited to foraging duties. As well, it has been observed that there is a minimum age, *a*_R_, before which bees cannot be recruited [39]. Behavioural maturation of hive bees is further regulated by a pheromone, ethyl oleate, that is produced by foragers [40] and has the effect of delaying the age at which bees are recruited to foraging duties. This so-called ‘social inhibition’ process reduces recruitment when the number of foragers in the colony is high [40]. We account for these biological processes by defining *u*(*a*) as

2.2 $$u(a)=\alpha {\left(\frac{a}{a+k}\right)}^{2}\left(1-\frac{\sigma }{N}\int (F_{S}+F_{I})\;da\right)H_{v}(a-a_{R}),$$

where *α* is the maximum rate of recruitment and *H*_v_(*a*−*a*_R_) is the Heaviside function such that recruitment cannot begin before age *a*_R_. Thereafter, the recruitment rate we are using increases sigmoidally with age. At age *k*, recruitment will be half the maximum rate of recruitment. 1/*σ* is the maximum proportion of bees that are foraging at any given time and *N* is the total number of bees given by

2.3 $$N=\int (H_{S}+H_{I}+F_{S}+F_{I})\;da.$$

The emergence of new hive bees is modelled as the left boundary condition to equation (2.1). This, along with other boundary conditions are described at the end of this section.

The second term on the right-hand side of equation (2.1) governs the disease dynamics within the hive. We approximate the transmission of disease as a mass action process and assume that, on average, hive bees and foragers transmit infection between or within classes at rate *β*. The total number of infected bees is given by

2.4 $$N=\int (H_{I}+F_{I})\;da.$$

The hive provides substantial safety for bees that are confined to it [11,30]. We therefore assume that the natural death rate of healthy hive bees is negligible compared to the rate of recruitment.

The equation governing the dynamics of infected hive bees is given by

2.5 $$\frac{\partial H_{I}}{\partial t}+\frac{\partial H_{I}}{\partial a}=\beta NH_{S}-u(a)H_{I}-d_{H}(a)H_{I}.$$

In contrast with their healthy peers, infected hive bees are at risk of dying due to disease at an age-dependent rate *d*_*H*_(*a*).

Susceptible foragers are recruited from susceptible hive bees, and suffer age-dependent natural death at rate *μ*(*a*). Their dynamics are therefore governed by

2.6 $$\frac{\partial F_{S}}{\partial t}+\frac{\partial F_{S}}{\partial a}=u(a)H_{S}-\mu (a)F_{S}-\beta NF_{S}.$$

Infected foragers can either be recruited from infected hive bees, or from susceptible foragers that have become infected. If we assume this class is subject to a disease-related death rate of *d*_*F*_(*a*), then their dynamics are governed by

2.7 $$\frac{\partial F_{I}}{\partial t}+\frac{\partial F_{I}}{\partial a}=u(a)H_{I}+\beta NF_{S}-(\mu (a)+d_{F}(a))F_{I}.$$

Food, *f*, is brought into the hive by both susceptible and infected foragers. Although it is likely that infected foragers would be less efficient at this task [41], for simplicity, we take an average rate of food intake, *c* (g/day/forager). Food is consumed by foragers and hive bees at an average rate *γ*. Therefore, the amount of food available at time *t* changes according to

2.8 $$\frac{df}{dt}=c\int (F_{S}+F_{I})\;da-\gamma N.$$

The above system of equations governing the dynamics of the colony is subject to the following boundary conditions:

2.9 $$\begin{array}{rl} H_{S}(0,t) & =LS,\\ H_{I}(0,t) & =F_{S}(0,t)=F_{I}(0,t)=0\\ \;\mathrm{and}\;\;\lim_{a\to \infty }H_{S}(a,t) & =\lim_{a\to \infty }H_{I}(a,t)=\lim_{a\to \infty }F_{S}(a,t)=\lim_{a\to \infty }F_{I}(a,t)=0. \end{array}\}$$

The first condition represents the emergence of new adult bees, where *L* is the daily egg-laying rate of the queen and *S* is a survivability function, which determines how many eggs survive to adulthood. The brood needs both sufficient food and sufficient care from the hive bees in order to survive [42]. Moreover, it has been shown that there is a range of ages within which hive bees will take on this care for the brood, a minimum age, *a*_*m*_, and a maximum age, *a*_*T*_ between 11 and 16 days old [32]. After this age, hive bees tend to transition to foraging duties, or possibly security or hive maintenance [33]. Therefore, we define the survivability function *S* as

2.10 $$S=\left(\frac{f}{b+f}\right)\left(\frac{\int_{a_{m}}^{a_{T}}H(a,t)\;da}{w+\int_{a_{m}}^{a_{T}}H(a,t)\;da}\right).$$

Here *b* is the amount of food required for half the eggs to survive to adulthood provided the brood has sufficient care, *w* is the number of care providers required for half the eggs to survive provided the brood has sufficient food, and *H*(*a*,*t*)=*H*_S_(*a*,*t*)+*H*_I_(*a*,*t*).

The dynamics described in this section relate to the main active season of the bee colony, which is defined as the time interval between the end of one winter and the beginning of the next.

### 2.2. Winter

The winter season is assumed to last 155 days or roughly five months, roughly corresponding to a humid continental climate [43]. Over winter, no new hive bees emerge (although some eggs are still being laid by the queen) and foragers return to the hive [33]. Accordingly, in boundary conditions (2.9) we set *L*=0, and in equations (2.1), (2.5)–(2.8) we set *u*(*a*)=0 and *c*=0. Owing to an extended lifespan of bees over winter [33], the death rate of hive bees is no longer negligible and is set to an average rate of

2.11 $$\mu_{w}=\frac{1}{180},$$

corresponding to an average lifespan over winter of six months [33]. All bees are performing the same function over winter (keeping the hive warm [44]); therefore, we set the natural death rate of foragers equal to that of the hive bees, *μ*(*a*)=*μ*_w_.

As winter ends, bees resume their normal age-related duties, and bees that were foraging before winter resume their roles as foragers. We assume that a 21-day transition takes place during which parameter values change linearly from end-of-winter to new active season values as follows:

2.12 $$\begin{array}{rl} \mu_{\mathrm{trans}}(a) & =\left(1-\frac{t}{21}\right)\mu_{w}(a)+\left(\frac{t}{21}\right)\mu (a),\\ L_{\mathrm{trans}} & =\left(\frac{t}{21}\right)L\\ \;\mathrm{and}\;\;S_{\mathrm{trans}} & =\left(\frac{t}{21}\right)S+\left(1-\frac{t}{21}\right)S^{*} \end{array}\}0\leq t\leq 21,$$

where

2.13 $$S^{*}=\left(\frac{f}{b+f}\right)\left(\frac{\int_{a_{m}}^{\infty }H_{S}(a,t)\;da}{w+\int_{a_{m}}^{\infty }H_{S}(a,t)\;da}\right),$$

that is, during winter hive bees of all ages may contribute to brood care. Note that new hive bees that emerge during the early spring come from eggs that were laid over the winter months, after completing phases of development to adulthood.

Equations (2.1), (2.5)–(2.8) form a system of integro-partial differential equations which we solved simultaneously to predict the population dynamics of a honeybee colony. The parameter values used in the solution are given in the electronic supplementary material, table S1.

In [45], the authors found that the age distribution in a colony is as follows: 41% of bees were one week old, 23% were two weeks old, 17% were three weeks old, 11% were four weeks old and 8% were five weeks old. Accordingly, the death rate *μ*_ex_(*a*) in our model was constructed to approximately match the experimental findings. To account for the whole range of ages and remain consistent with the five weeks of study in [45], we break the age range into five cohorts of 10 days each. The comparison between experimental data and our model is summarized in table 1. The death rate that best matched the observed data is defined by

**Figure 1.**
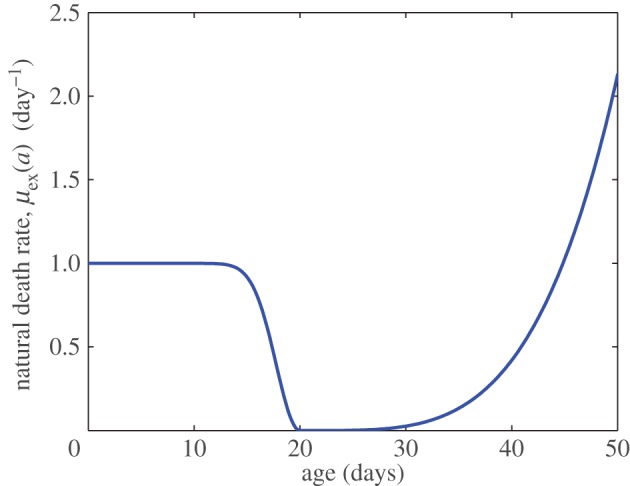
Death rate distribution which provides the best fit to experimental results observed in [45]; parameter *C*_ex_=0.42.

2.14 $$\mu_{\mathrm{ex}}(a)=\left\{ \begin{array}{ll} 1-e^{{\left(a-20\right)}^{2}/10} & a\leq 20\\ C_{\mathrm{ex}}{\left(\frac{a-20}{20}\right)}^{4} & a>20, \end{array}\right.$$

where parameter values were set such that the percentage of bees in their first week of life matches the experimental data. This death rate is relatively high for very young (approx. <10 days) or very old bees (approx. >40 days), as shown in figure 1. We see that the drop in population density after week 1, as observed in [45], can be explained by the high death rate of young foragers.

**Table 1.** Comparison of experimental results with model results. Standard deviations for experimental results are given; *z*-scores computed from experimental means and standard deviations are given in parentheses next to model values.

cohort	experimental values [45]	model values, *μ*_ex_(*a*)	model values, *μ*(*a*)
1	41±6.4%	41% (0)	41% (0)
2	23±5.1%	25% (0.39)	30% (1.37)
3	17±3.7%	18% (0.27)	16% (0.27)
4	11±2.2%	11% (0)	7% (1.81)
5	8±1.3%	5% (2.31)	6% (1.54)

We performed sensitivity analysis (see the electronic supplementary material, figure S1) and found that our results are insensitive to the details of the death rate distribution. We therefore use a simplified version of the natural death rate distribution

**Figure 2.**
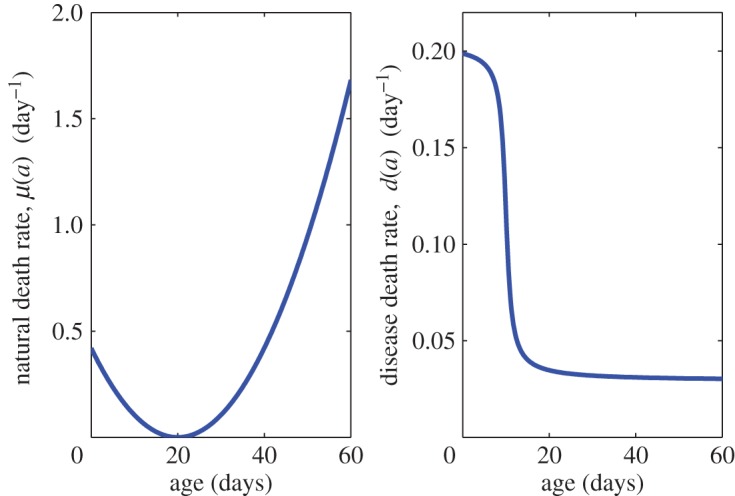
The natural death rate, *μ*(*a*), and an example of disease-related death rate, *d*(*a*), motivated by deformed wing virus, which predominantly affects young bees [46]. During the active season, honeybees live an average of five to seven weeks [33], thus we define our death rates until the age of 60 days. Beyond this age few, if any, bees are still alive and we set their natural death rate as μ(a>60)=max(μ(a)).

2.15 $$\mu (a)=C\frac{{\left(a-20\right)}^{2}}{400},$$

to make the mathematics more tractable. This death rate is plotted in figure 2, and also compared with the experimental results in table 1, showing good agreement.

The disease-related death rate

2.16 $$d(a)=-\frac{K_{1}}{\pi }\tan^{-1}(a-10)+K_{2}$$

is motivated by deformed wing virus [46] that disproportionately affects young bees. The constants *C*, *K*_1_ and *K*_2_ are chosen such that both the natural and disease-related death rates are non-negative and have an average of 0.14 per day as in [13]. In other cases, we assume the disease affects all bees uniformly, i.e. *d*(*a*)=*d*. The effects of different death rate distributions are shown in the electronic supplementary material, figures S4–S12. The results are qualitatively the same.

Electronic supplementary material, figure S1, shows the age distribution within a disease-free colony under the natural death rate *μ*(*a*) shown in figure 2. This is used as a baseline distribution for all simulations that begin from equilibrium. We predict from this distribution that the mean forager age of a healthy colony is approximately 25 days, in agreement with [33,47].

## 3. Results

**Figure 3.**
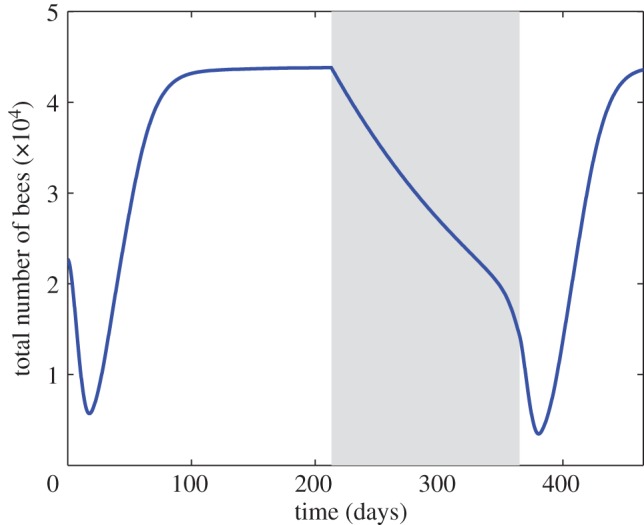
Time course of total bee population in a disease-free colony. Shaded area denotes winter, during which no brood is produced. We see that the aged bees that restart the colony post-winter create an added risk to the colony, because of their relatively high death rate, until new brood is able to hatch and contribute to population rebound. The post-winter dip seen in the figure has been referred to in the literature as ‘spring dwindle’ but its dynamics have not been previously described.

Figure 3 shows the effects of changing seasons on the number of bees in a colony in the absence of disease. The seasonal shift back to normal operating conditions is seen to bring with it a drop in the total number of bees as the colony emerges from winter. It is obvious from these results that a longer winter may have a deleterious effect on the colony since the effects of winter are largely negative. This is because the surviving bees are now much older and are subject to a higher natural death rate, while a new generation of younger bees has not yet emerged to replace them. This seasonal drop in the bee population, which has been referred to as ‘spring dwindle’ [34–37], is discussed further in the next section. The colony dynamics that produce the phenomenon have not been fully understood in the past because these dynamics emerge only when the age distribution within the colony is considered.

**Figure 4.**
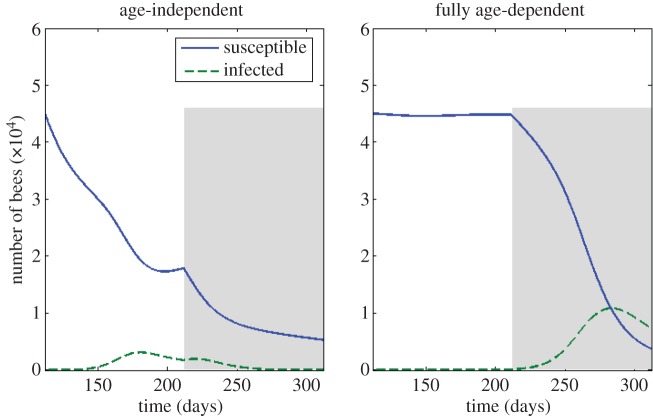
The effect of age distribution on the dynamics of disease within the bee colony. The blue line represents the total number of susceptible bees and the green dashed line represents the total infected bees. The shaded area denotes winter. Disease begins with a single infected forager at *t*=112. We use the death rates given in figure 2. The two time courses show that the two models have significant differences in their predictions when simulating disease dynamics in the colony.

Our next objective is to compare the dynamics of the age-structured honeybee colony model with the dynamics of the age-independent model such as that presented in [13]. To give the comparison a measure of equivalency, the same value of the basic reproduction number is used in both cases, namely *R*_0_=1.43, calculated using the formulae derived in [14]. Figure 4 shows that an age-independent model makes substantially different predictions about the timing and the severity of disease within a bee colony. This is further explored in the electronic supplementary material, figure S3, where we demonstrate that an age-dependent recruitment function both lowers the severity of a disease and delays the timing of an epidemic, while age-dependent death rates, *μ*(*a*) and *d*(*a*), trade off the two effects.

**Figure 5.**
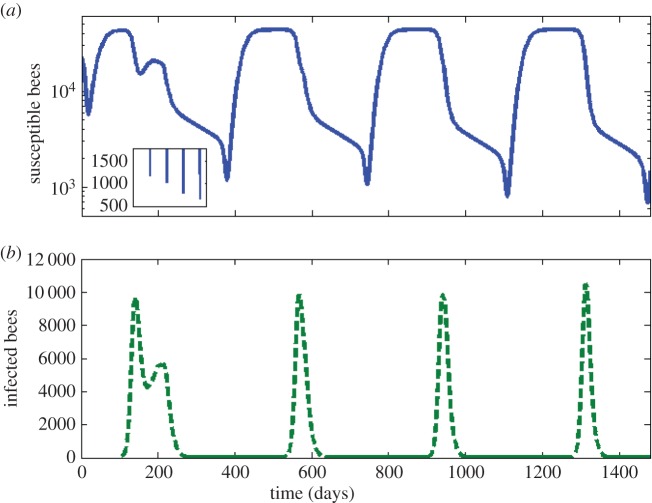
Time course of the bee population over 4 years. (*a*) The total number of susceptible bees, *H*_S_+*F*_S_ and (*b*) the total number of infected bees, *H*_I_+*F*_I_. An infection is introduced into a colony at equilibrium through a single-infected forager at time *t*=0. The inset shows the minimum population size of susceptible bees during the spring dwindle following each winter. There is a clear decrease in this minimum year after year, thus the risk of colony collapse increases year after year. The ultimate collapse of a bee colony may therefore be the consequence of events long past.

The vulnerability of a bee colony in early spring is explored further in figure 5 which illustrates that an infection which is endemic in a colony during one active season and one winter may pose the greatest risk to the colony during the small window in which the hive is recovering from the winter months. Moreover, the colony may suffer much greater losses after its second, third and fourth winters with disease (figure inset).

**Figure 6.**
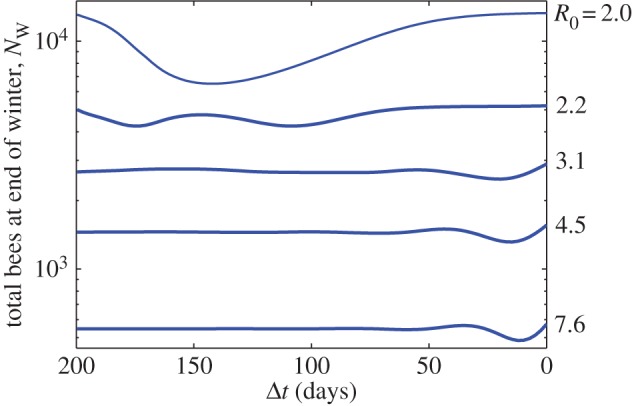
Number of bees at the end of winter as a function of the time interval, Δ*t* between the onset of disease and the beginning of winter, based on various values of the basic reproduction number, *R*_0_. The minima show times during which the colony is most vulnerable due to the combined effects of winter and disease. The colony is started from the equilibrium age distribution shown in the electronic supplementary material, figure S1. Infection is introduced at time *t*=200−Δ*t* via one infected forager. The total number of bees is calculated at the end of winter. *R*_0_ values are calculated using the expression derived in [14].

The time interval between the beginning of an infection and the onset of winter is an important determinant of the health of the colony at the end of winter, as illustrated in figure 6. The figure shows that the number of bees surviving to the end of winter, *N*_W_, depends on when the infection begins relative to winter. In the figure, the infection begins from a single-infected forager Δ*t* days before winter. The time interval between the end of one winter and the beginning of the next is taken to be approximately 200 days (approx. seven months). The timing of the infection is seen to produce a point of highest vulnerability (local minimum in population size) which depends on the severity of the disease as represented by the basic reproduction number.

**Figure 7.**
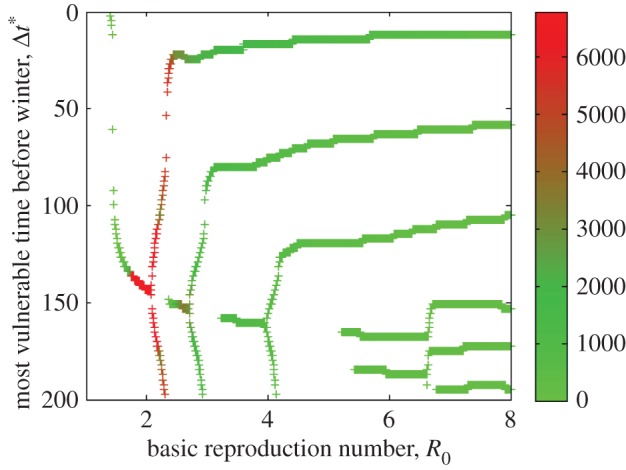
The timing and depth of points of highest vulnerability as a function of the basic reproduction number, *R*_0_. The colour bar reflects the depth of the minima seen in figure 6 (see text for details). Red indicates the most severe combinations of *R*_0_ and Δt*, where Δt* is the value of Δ*t* in figure 6 at which a local minimum occurs.

The timing and depth of the points of highest vulnerability seen in figure 6 are tightly coupled to the time at which an infection peaks within the colony, which in turn is related to *R*_0_. Figure 7 shows this relationship, estimating the time of greatest risk to the colony, Δt*, with respect to the onset of winter, based on the basic reproduction number. Of course, for high values of *R*_0_ the colony is likely to suffer substantial losses regardless of when a disease occurs. In figure 6, we see, for example, that for *R*_0_=2.0, Δt* is unique, but when *R*_0_=2.2 there are two Δt* values producing the observed bifurcation in figure 7. To measure the relative significance of these minima, we use the metric $\max(N_{W})-\min(N_{W}),$ where the maximum and minimum are computed for each value of *R*_0_, over the range of possible Δ*t*. The larger the value of this metric, the greater the significance of the time of onset of disease, Δ*t*.

## 4. Discussion

Our results demonstrate that the age structure that is inherent to all honeybee colonies has critical effects on colony survival that are not captured by previously explored age-independent models. Most notably, an age-dependent recruitment rate has a profound impact on the dynamics, severity and spread of an infection in a bee colony. In fact, our results predict that age polyethism in a honeybee colony has the beneficial effect of impeding the spread of infection within the colony. Thus, in practice, any disturbance in the natural age structure of a bee colony may also increase the severity of an infection within the hive. Results in the electronic supplementary material, figure S3, indicate that the natural age distribution within a colony creates a delay in the speed of spread of an infection. Again, this suggests that any intervention that would alter the natural age distribution may have the adverse effect of increasing the spread of infection within a hive.

Our explicit treatment of the colony age distribution also revealed the dynamics underlying the frequently observed phenomenon of ‘spring dwindle’ in which the total number of bees within a colony weakens rapidly immediately after the end of winter. While the phenomenon is generally suspected to be due to various stressors that occur during winter [35–37], our results suggest that declining spring populations are a natural consequence of honeybee colony dynamics, leading to seasonal vulnerability during which the colony may be particularly susceptible to other hazards. This post-winter dip is not captured by age-independent models because the phenomenon is due predominantly to the ageing of the bees over the winter months and the time required to replace them by younger bees in the early spring.

Simulations of colony dynamics over multiple winter seasons demonstrated that endemic diseases may weaken the colony year after year, increasing the risk of colony collapse during spring. Thus, the eventual collapse of a bee colony may be due to the compounded effects of an infection over several years.

The time interval between the onset of an infection and the beginning of an approaching winter is a critical determinant of the ultimate course and consequence of the disease within a bee colony. As illustrated in figures 6 and 7, this effect is most important for infections with a basic reproduction number close to 2. In this case, the colony is particularly vulnerable in the early spring, approximately five months before the onset of winter.

In conclusion, age structure is an inherent property of honeybee colonies, determining the division of labour and defining how age-related events such as disease and death unfold within the colony. Our results suggest that age structure should be a key consideration in future studies of honeybee population dynamics, and that accurate models of age structure will be necessary if we are to understand, and ultimately reverse, the increases in colony collapse observed in recent years.

## Supplementary Material

Age structure is Critical to the Population Dynamics and Survival of Honey Bee Colonies: Supplementary Material

## Data accessibility

This study has no data.
