# Supplementary material for: Age structure is critical to the population dynamics and survival of honeybee colonies
Source: R Soc Open Sci. 2016 Nov 16;3(11):160444. doi: 10.1098/rsos.160444 (PMC5180125; doi:10.1098/rsos.160444)
Supplement: Age structure is Critical to the Population Dynamics and Survival of Honey Bee Colonies: Supplementary Material [file rsos160444supp1.pdf]

Age structure is Critical to the Population Dynamics and Survival of Honey Bee Colonies

M.I. Betti[1], **L.M. Wahl**[1], **M. Zamir**[1,2,†]

[1]Department of Applied Mathematics

[2]Department of Medical Biophysics

Western University

London, Ontario, N6A 5B7

CANADA

[†]corresponding author: zamir@uwo.ca

October 15, 2016

Supplementary Material

Table 1: Parameter values and source references.

L	maximum rate of egg laying	2000 eggs/day	[5]
w	number of hive bees for 50% egg survival	5000 bees	[5]
a_m	age at which hive bees begin brood care	3 days	[7]
a_T	age at which hive bees end brood care	11 days	[7]
a_R	minimum recruitment age	4 days	[2]
k	age at which rate of recruitment is 25% of max.	10 days	
α	maximum rate of recruitment	1 /day	
$\frac{1}{\sigma}$	maximum fraction of colony that can be foraging	$\frac{1}{3}$	[5]
$\mu(a)$	natural death rate of foragers (summer)	Figure 1	[1]
μ_w	natural death rate of foragers and hive bees (winter)	1/180 /day	[7]
b	mass of food stored for 50% egg survival	500 g	[4]
c	food gathered per day per forager	0.1 g /day / bee	[6]
γ	daily food requirement per adult bee	0.007 g /bee	[4]
$d_H(a)$	death rate of hive bees due to infection	0.14 /day or Figure 1	[3]
$d_F(a)$	death rate of hive bees due to infection	0.14 /day or Figure 1	[3]
β	disease transmission rate	variable	

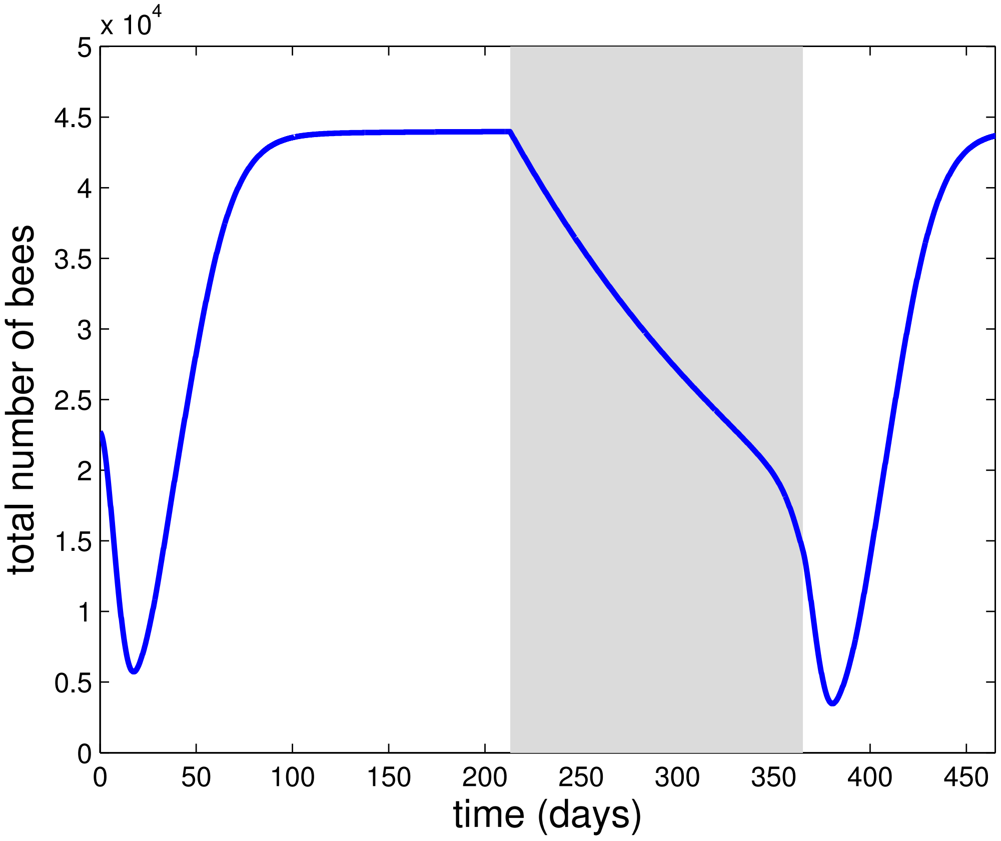

Figure S1: Time course of total bee population in a disease-free colony. Shaded area denotes winter, during which no new hive bees are produced. Here we use more realistic food intake $c = 0.5$ and consumption rates, $\gamma = 0.07$ [37]. We see that the dynamics of the colony are not sensitive to the food intake and consumption rates.

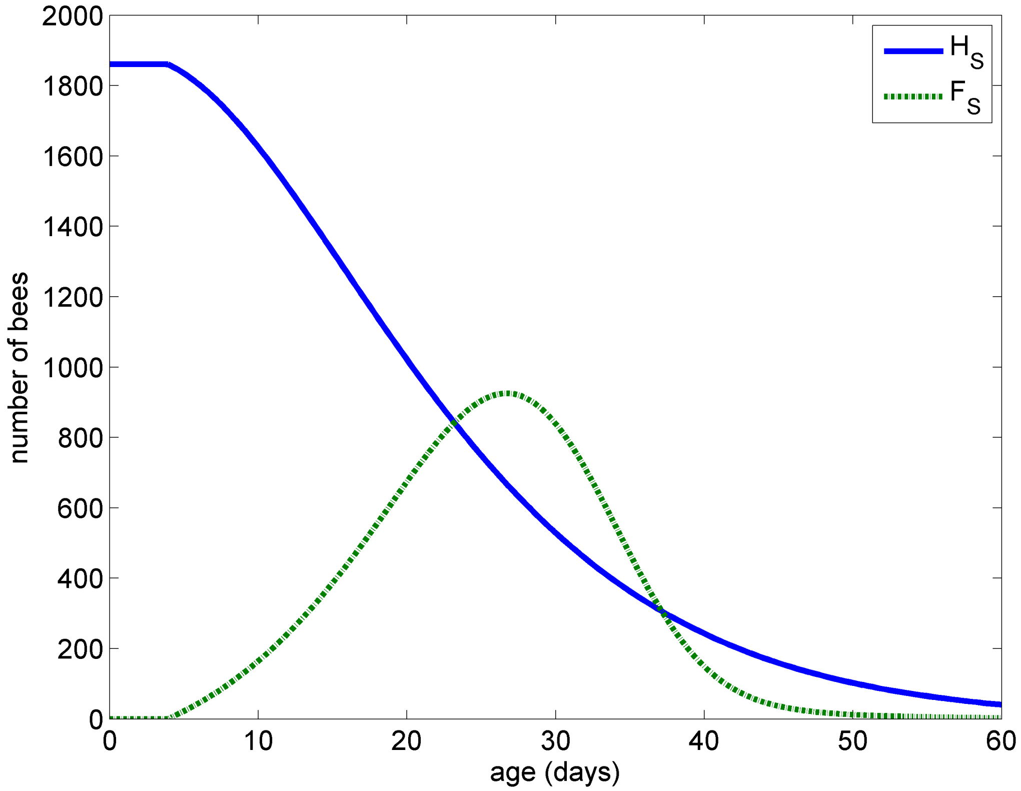

Figure S2: Age distribution of a healthy colony at equilibrium during the active season, based on the natural death rate, $\mu(a)$ presented in Figure 1.

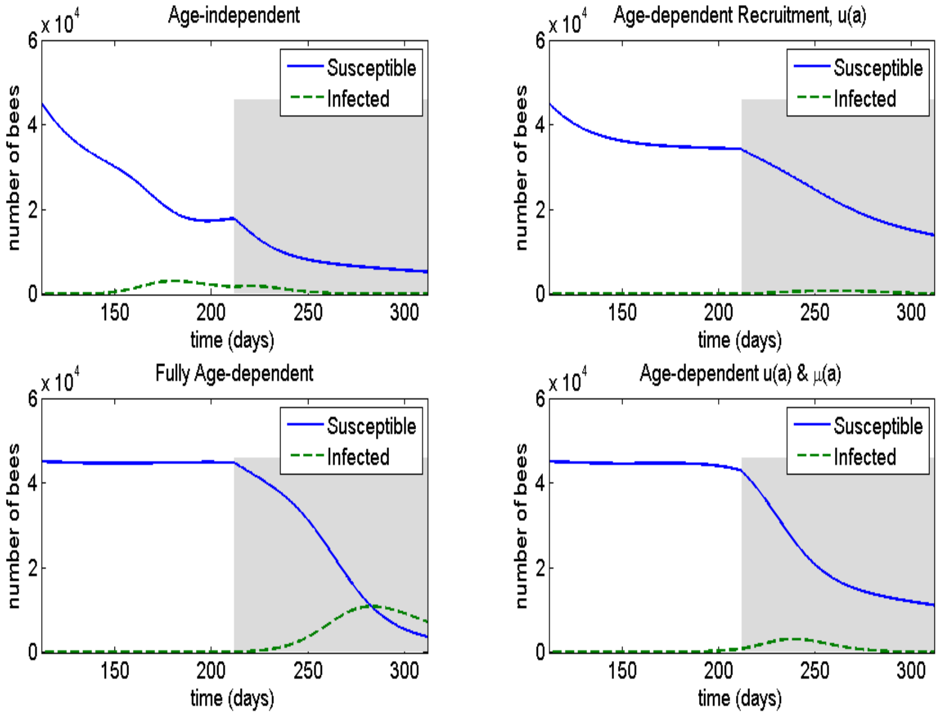

Figure S3: Population dynamics with increasing effects of age-dependent parameters: clockwise from top-left (age-independent model) to bottom-left (fully age-dependent model).

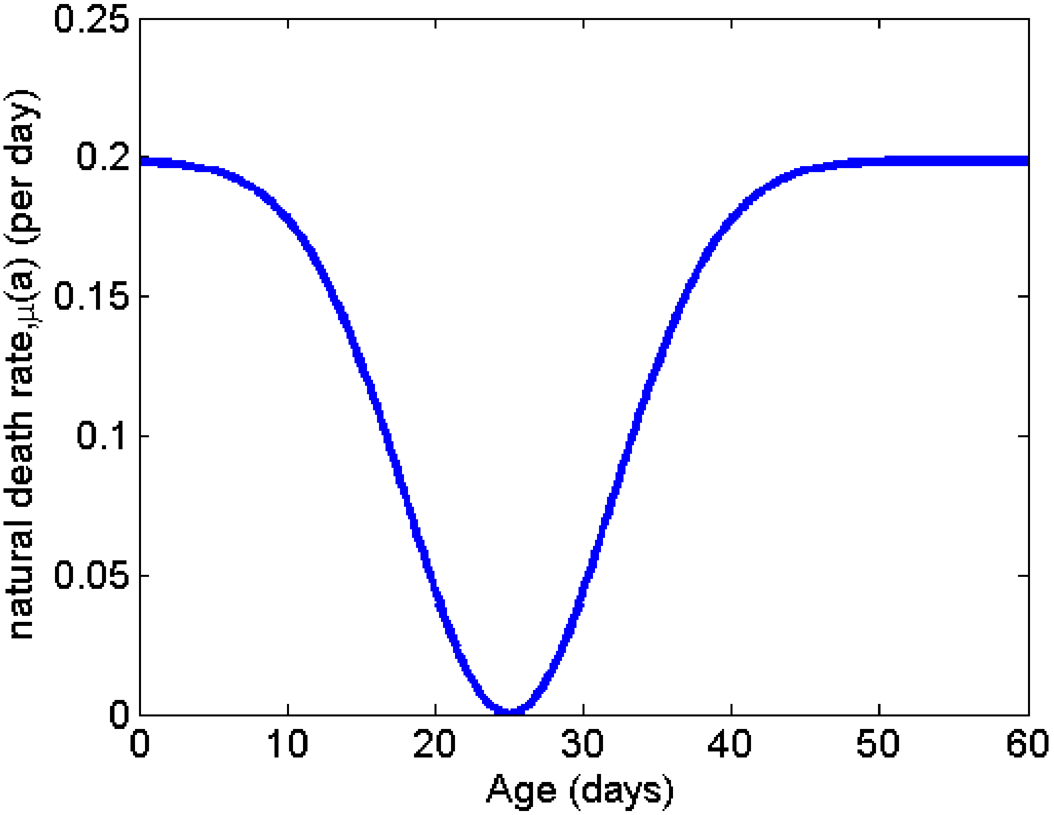

Figure S4: An inverted Gaussian natural death rate, $\mu(a) = A\mathrm{e}^{-(a-25)^2/B}$ where A and B are chosen such that the average death rate is $\mu_{avg} = 0.14$ per day. Results based on this death rate are shown below.

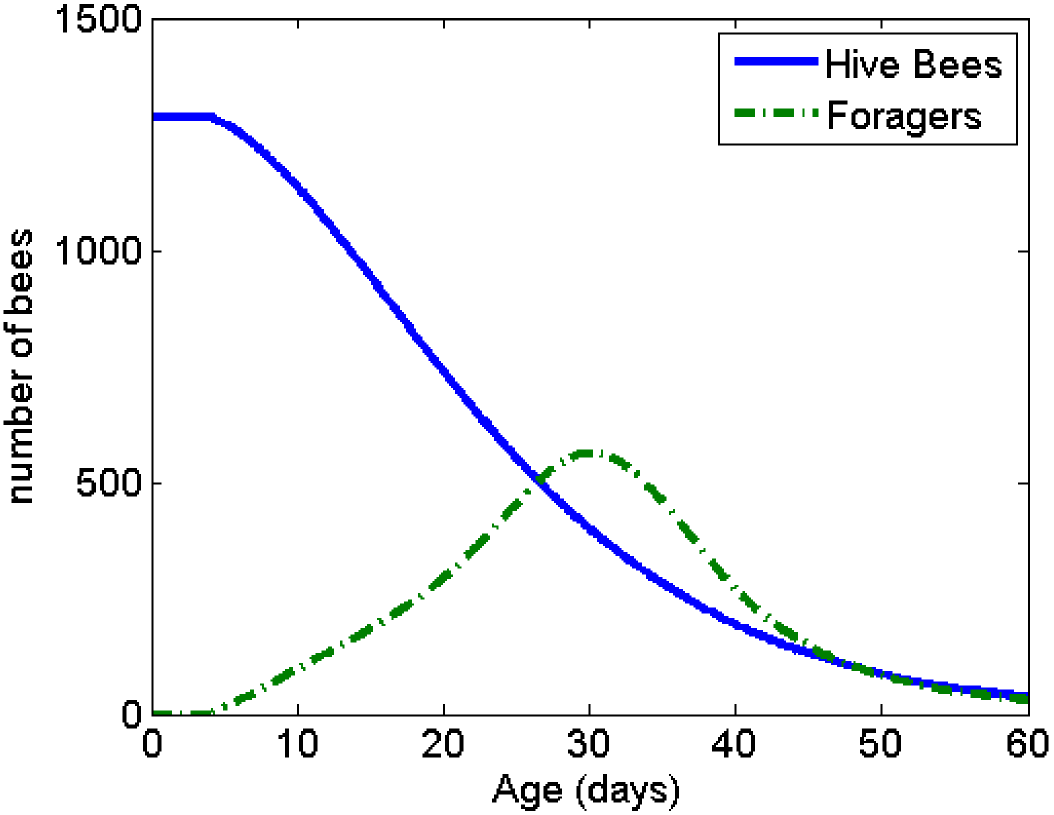

Figure S5: Equilibrium age distribution of healthy colony under the natural death rate shown in Figure S4.

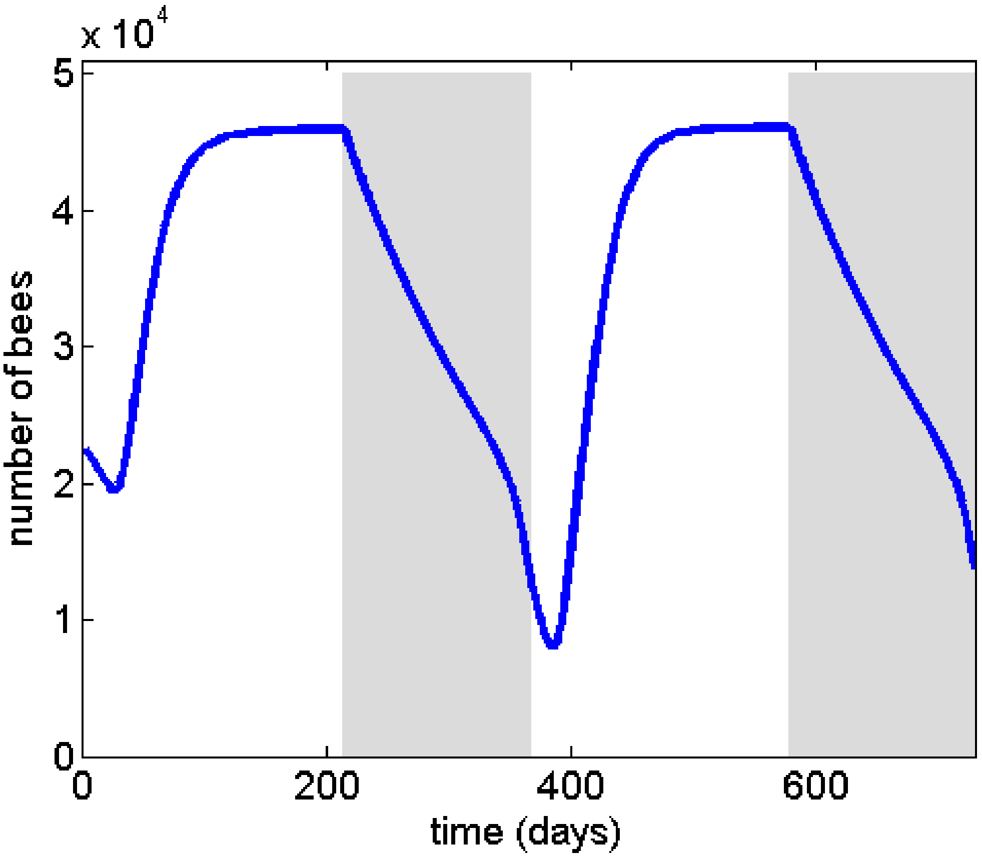

Figure S6: Time course of disease-free colony based on natural death rate $\mu(a)$ shown in Figure S4.

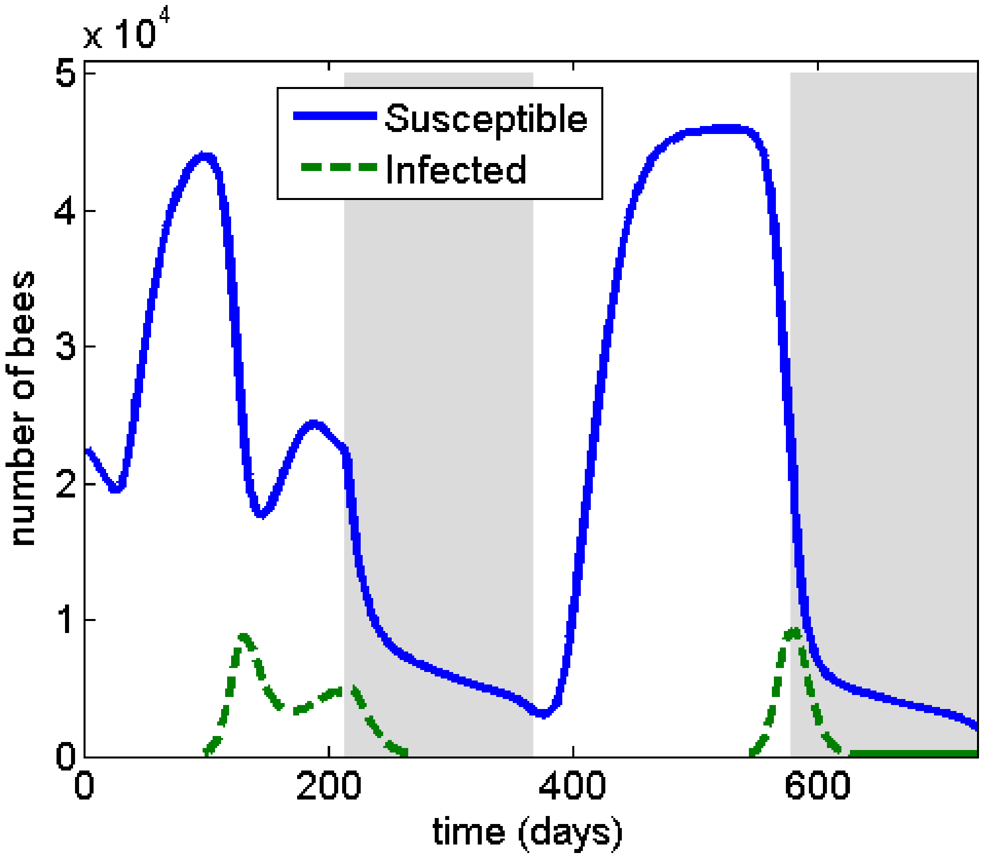

Figure S7: Time course of infected colony based on natural death rate $\mu(a)$ shown in Figure S4 and a constant disease death rate $d(a) = d = 0.14$ per day.

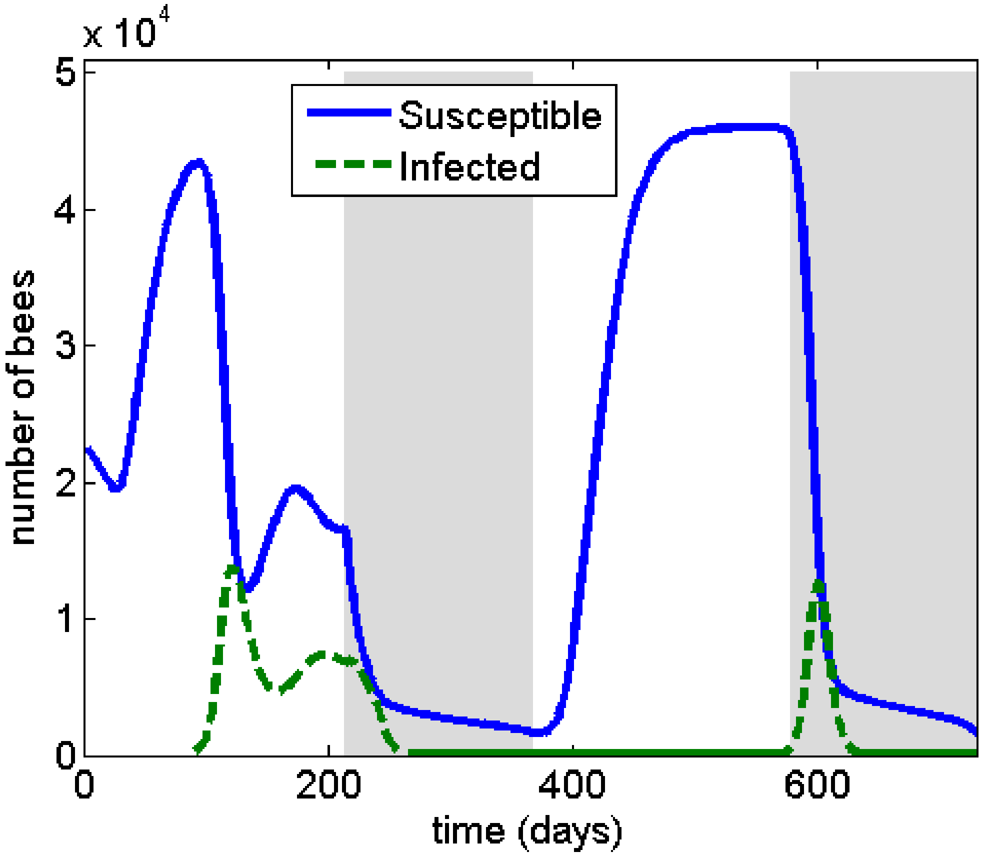

Figure S8: Time course of infected colony based on natural death rate $\mu(a)$ shown in Figure S4 and a disease death rate $d(a) = \mu(a)$.

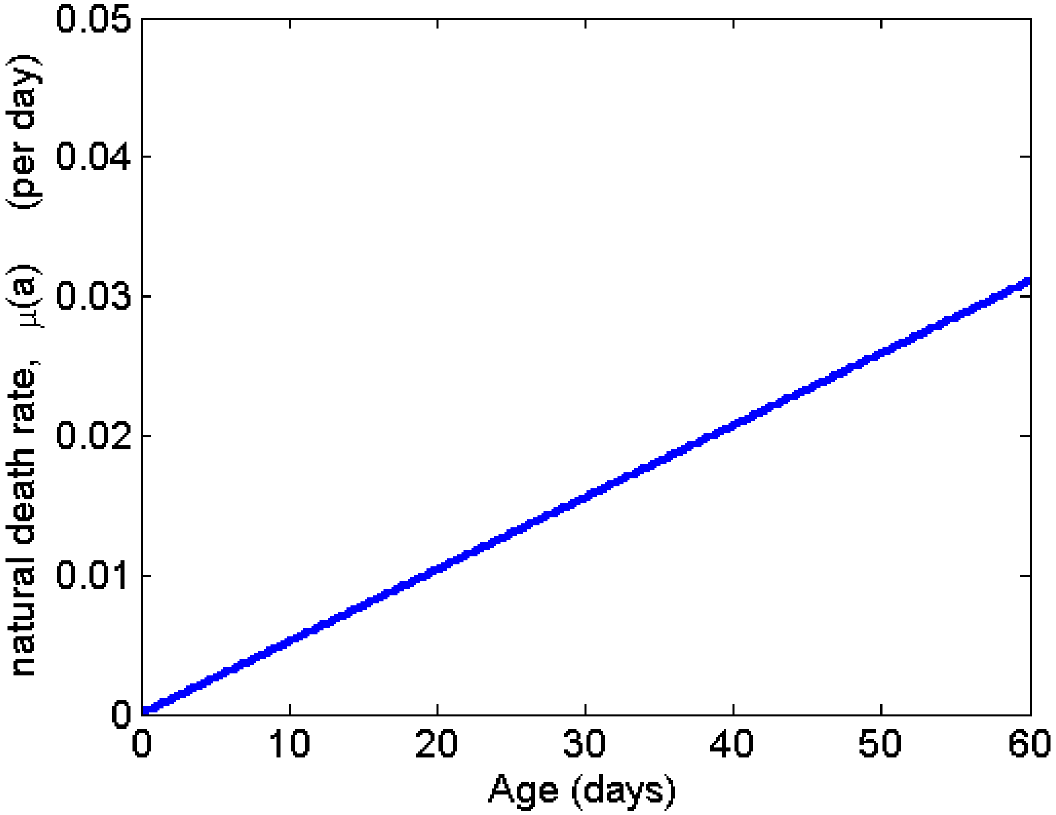

Figure S9: An admittedly unrealistic linear natural death rate $\mu(a) = Aa$ where A is chosen so once again the average is 0.14 per day. We show this death rate to illustrate that the qualitative results of our model hold in even with drastically different parameter choices.

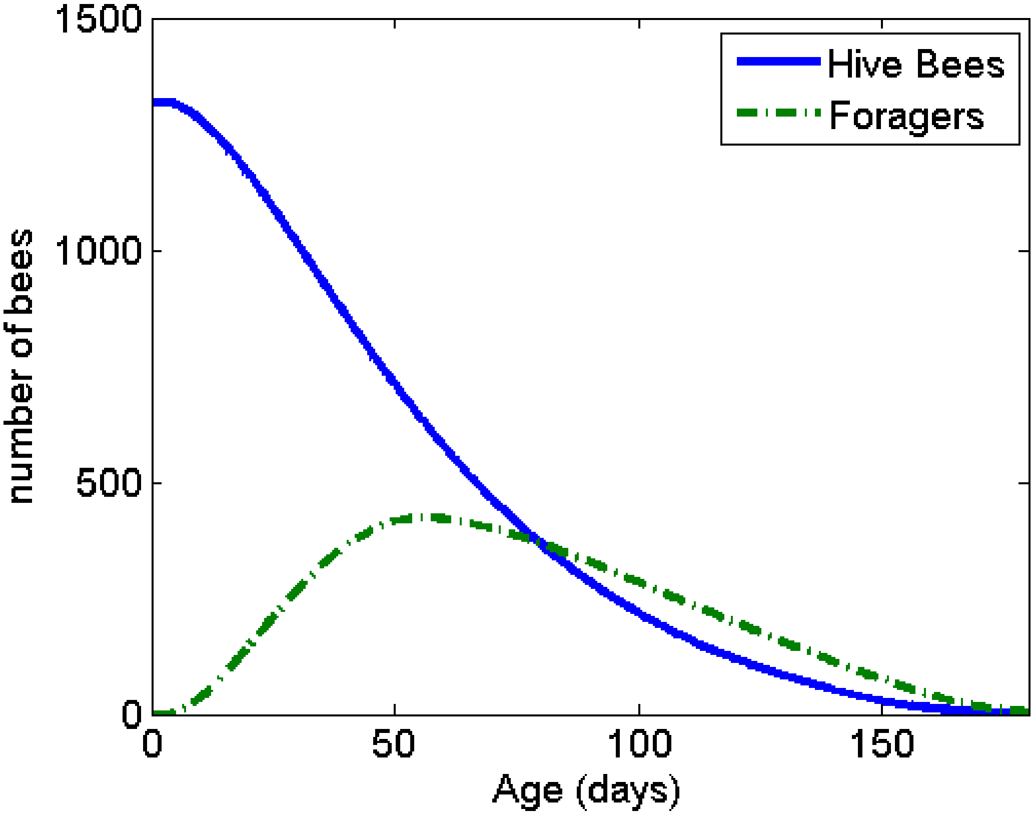

Figure S10: Equilibrium age distribution of healthy colony under the natural death rate shown in Figure S9. Note that the linear death rate leads to an unrealistically long lifespan.

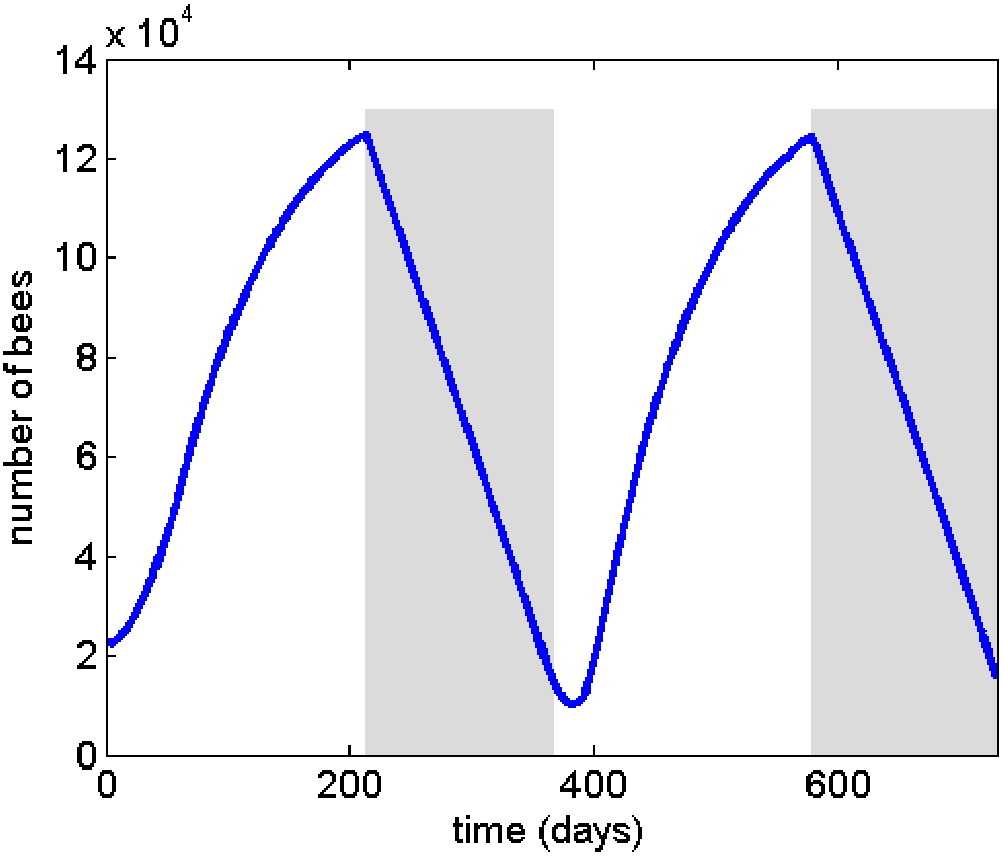

Figure S11: Time course of disease-free colony based on natural death rate $\mu(a)$ shown in Figure S9. Despite the unrealistically large colony size and absurdly long lifetime of a bee, we still observe the spring dwindle as a natural phenomena.

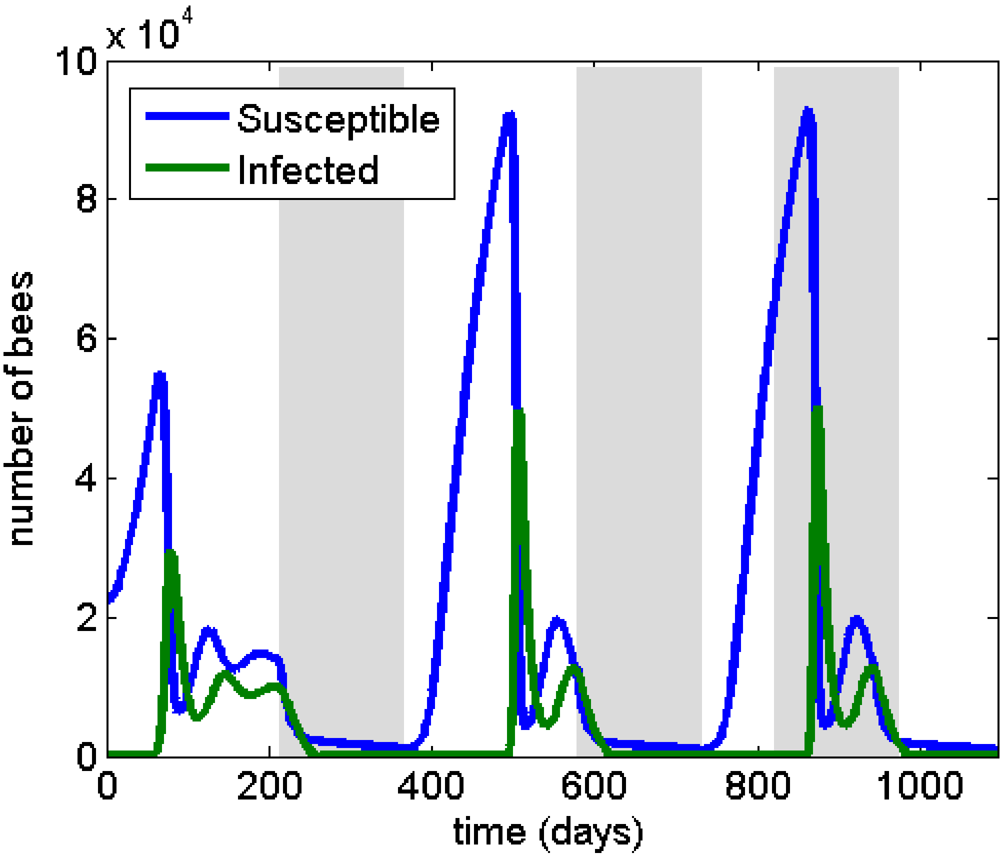

Figure S12: Time course of infected colony based on natural death rate $\mu(a)$ shown in Figure S9 and a Gaussian disease-related death rate, $d(a)$. Note that the infection continues to spike years after the initial infection.
